# Socio-demographic characteristics and pre-hospital care of children with circulatory failure in a children’s emergency room in southern Nigeria

**DOI:** 10.11604/pamj.2021.40.65.30003

**Published:** 2021-09-28

**Authors:** Moses Temidayo Abiodun, Wilson Ehidiamen Sadoh

**Affiliations:** 1 Department of Child Health, University of Benin Teaching Hospital, Benin City, Edo State, Nigeria,; 2 School of Medicine, University of Benin, Benin City, Edo State, Nigeria

**Keywords:** Circulatory failure, socio-demographics, prehospital care

## Abstract

**Introduction:**

circulatory failure is a major childhood emergency. Several disease-related and patient-related factors can predispose children to shock. Early detection of such factors will improve its prevention, management and outcome. This study aimed to evaluate the incidence, socio-demographic characteristics and pre-hospital care of children presenting with circulatory failure (shock) in children´s emergency room (CHER).

**Methods:**

this study adopted cross-sectional design in CHER of the University of Benin Teaching Hospital, Nigeria, from October 2018 to March 2019. Data were collected using a semi-structured questionnaire eliciting demography, socio-economic status, pre-hospital care and presence of shock. In a sub-analysis, multiple logistic regression identified variables that are independently associated with circulatory failure in the participants, using adjusted odds ratio (OR) and 95% confidence intervals (CI).

**Results:**

a total of 554 acutely-ill children participated in the study. Their median age was 60 (IQR: 24-132) months. Shock was present in 79 (14.3%) of the children on arrival at CHER. Children referred from private clinics were more likely to arrive CHER in shock compared to those coming directly from home (OR = 2.67, 95%CI: 1.07-6.69; p = 0.036) while children from lower socio-economic class families presented more frequently with shock than those from higher class (OR = 14.39, 95% CI: 2.61-79.44; p = 0.002). Also, children that received oral rehydration solution as pre-hospital care seemed more likely to present with shock in CHER (OR = 6.63, 95% CI: 2.15-20.46; p =0.001).

**Conclusion:**

quality of pre-hospital care and parental socio-economic status influence the presence of shock in children seen at the emergency unit. Focused health education and prevention of finance-related delays in emergency care are needed.

## Introduction

Circulatory failure or shock is a common emergent morbidity encountered in acutely-ill children in the emergency room. It is a state of acute energy failure at the cellular level due to inadequate glucose and substrate delivery, hypoxemia or mitochondrial failure [1,2]. In a prospective study by Carcillo *et al*. [3], shock was present in 37% of children transferred to 5 regional hospitals for tertiary care. Also, shock can develop in a child while on admission. Fisher *et al*. [4] reported that clinical signs of shock developed in the emergency department (ED) after initially presenting without clinical signs of shock in 14% of cases in their series. Shock constitutes a life threatening illness in a child. The resultant anaerobic respiration leads to elevated serum lactate which is a poor prognostic factor in severe illness [5,6]. When shock is not recognized and reversed promptly, it leads to widespread ischaemic damage in all organ systems in the body, including the gastrointestinal system where there can be translocation of bacteria into the blood stream, contributing to systemic inflammatory response syndrome (SIRS), irreversible shock and multi-organ dysfunction syndrome (MODS) in affected children [7,8].

There are several disease-related and patient-related factors that can predispose children to circulatory failure [9,10]. Prompt recognition of these characteristics can facilitate case-specific evaluation and treatment of at-risk children. Although children have a high total body water, they are predisposed to shock because of their relatively increased basal metabolic rate and insensible water loss as well as low renal concentrating ability [11]. Also, chronic illnesses, hypothermia and malnutrition worsen severity of shock leading to presentation in emergency units in moribund conditions [12]. In addition, caregiver-related factors can influence the incidence and outcome of circulatory shock. Low parental educational and socio-economic status leads to poor health-seeking behavior which contributes to the high mortality rates in childhood emergencies in Nigeria and many sub-Saharan African countries [13,14]. Likewise, home remedies like herbal concoction with potential severe adverse reactions can contribute to deranged homeostasis and shock in children [15,16]. Hence, some baseline patient characteristics alongside potentially-modifiable factors contribute to the occurrence and prognosis of circulatory failure in acutely ill children. Considering the foregoing, the burden of circulatory failure in a practice setting can be reduced if the predisposing factors are identified and prevented in at-risk children. We hypothesized that some socio-demographic characteristics are associated with circulatory failure in acutely-ill children seen in our emergency room. Also, we aimed to identify the incidence, pre-hospital therapies and practices that predispose to shock in the setting.

## Methods

**Study design and setting:** this study used a descriptive, cross-sectional design. It was undertaken in the Children Emergency Rooms (CHER) of the University of Benin Teaching Hospital (UBTH), in southern Nigeria, from October 2018 to March 2019.

**Participants:** these were children who participated in a larger study evaluating treatment and outcome of shock in CHER at the centre. They were aged 1 month to 18 years and were admitted into CHER with critical illnesses based on the inclusion criterion.

**Inclusion criteria:** all children aged between 1 month and 18 years with signs/ symptoms of critical illness, and whose parents consent to the study. Critical illness was defined as the presence of any acute life-threatening disorder in the child requiring urgent interventions to prevent death [17].

**Exclusion criteria:** all children less than 1 month old who are admitted directly into the neonatal intensive care unit. Also, children who do not meet the standard clinical definition for critical illness during the study period were excluded.

**Sample size determination:** the minimum sample size (N) was calculated using the qualitative variable formula for cross-sectional study [18]:


$$N=Z_{1^{2}-\alpha }\left(P\right)\left(1-P\right)/d^{2}$$

where, Z_1-α_=normal standard deviation for confidence level of 95%=1.96; P=proportion of children with cardiovascular shock; we assumed an incidence of 50% for shock in critically-ill children (since the actual incidence in the setting was not known), a 95% confidence interval and a sample error of 5% [18]; D = margin of error to be tolerated (commonly fixed at 5% or 0.05). This was adjusted for a 10% non-response rate. A total of 554 children were recruited during the study period.

**Sampling strategy:** all children admitted into CHER with features of critical illnesses based on the inclusion criterion were purposively selected; they were recruited into the study if their parents or caregivers consented. This was a total population study of all eligible children during the study period.

**Data collection:** data were collected using a semi-structured researcher-administered questionnaire eliciting descriptive variables (demography, socio-economic status, prehospital care) and the presence of circulatory failure in the participants. Their socio-economic classification was based on their parental educational levels and occupations [19]. Blood pressure measurements was done using standard procedures with Mercury sphygmomanometer and appropriate cuffs [20]. Circulatory failure was defined based on WHO criteria (cold extremities, prolonged capillary refill >3 seconds, and fast and weak pulse) or the presence of hypotension (systolic BP < 70mmHg in infants or <70 +2n, where n = age in years for under-ten children) [11,17].

**Statistical analysis:** data was analysed using descriptive and inferential statistics. Categorical variables were described using frequencies and percentages while continuous variables such as age, duration of illness were described using means and standard deviations. The incidence of circulatory failure was computed based on the proportion of the participants affected at presentation. Bivariate analysis (Pearson chi-square; Mann-Whitney U test) was done to detect any significant association between the descriptive variables and circulatory failure. Variables (source of patient, maternal educational level, social class and prehospital care) that were significant (p<0.05) on binary analysis were then subjected to multivariate logistic regression to identify those that are independently associated with circulatory failure in the participants, using adjusted odds ratio (aOR) and 95% confidence intervals (CI). The level of significance was set at p < 0.05. The data analysis was carried out using the IBM Statistical Package for Social Sciences (SPSS) version 26.0 for windows.

**Ethical consideration:** Ethical clearance was obtained from the Research and Ethics Committee of the College of Medical Sciences, University of Benin (REC Approval Number: CMS/REC/2018/020). Permission of the Unit Head CHER was sought and informed consent was obtained from the parents/guardians of the children.

## Results

**Baseline characteristics of all study participants:** a total of 554 acutely-ill children took part in the study. They had a median age of 60 (IQR: 24-132) months, mean weight 16.3 ±13.6 kg, mean height 90.8 ± 33.2 cm and their female to male ratio was 1: 1.2. Over a half (53.8%) of the caregivers (mothers) had secondary level of education while 42.2% and 34.6% of the families were in lower and middle socioeconomic classes respectively. Most of the children 363 (80.0%) presented to the emergency room coming directly from home; the rest of them were referred equally from both private and public hospitals. Shock was present in 79 (14.3%) of the children on arrival at CHER. Their median (IQR) duration of illness before admission was 4 (2-7) days and the pre-hospital care received included administration of ORS (7.4%) and herbal concoction (2.2%); further details are shown on Table 1.

**Table 1:** baseline characteristics of study participants, recruited from the Children Emergency Room of the University of Benin Teaching Hospital (Nigeria), from October 2018 to March 2019 (N=554)

Characteristics	Frequency	Percentage
**Age**		
<1	219	40.2
1-5	194	35.6
>5	132	24.2
Median age [IQR] (months**)**	60 [24-132]	
**Maternal level of education**		
None	68	15.6
Primary	134	30.7
Secondary	235	53.8
Tertiary	0.0	0.0
**Religion**		
Christianity	523	94.4
Islam	15	2.7
Jehovah witness	4	7
Others	12	2.2
**Source of patient's referral**		
Private	46	10.1
Public	45	9.9
Home	363	80.0
**Shock at presentation**		
No	475	85.7
Yes	79	14.3
**Pre-hospital care**		
None	324	58.5
ORS	41	7.4
Herbal concussion	12	2.2
Others*	177	31.9
**Social class**		
Upper	113	23.2
Middle	169	34.6
Lower	206	42.2

IQR = Interquartile range; *Over-the-counter (OTC) drugs and medications received in referring health facilities

**Comparison of characteristics between the children with and without circulatory failure:** comparison of characteristics between the acutely-ill children with and without cardiovascular shock showed that their sources of referral were significantly different (p = 0.011); likewise, there was significant difference between their maternal level of education (p = 0.001), pre-hospital care (p <0.001) and socio-economic classes (p < 0.001). None of the mothers who gave information on their educational level had tertiary education. The median age of children with shock was similar to that of their unaffected counterparts, 66 (24-132) vs. 60 (24-120) months respectively (p = 0.824). Also, there was no gender predilection for shock (p= 0.701) and the durations of their illnesses before admission were similar 4 (2-7) vs. 4 (2-7) days (p = 0.552). Further comparative data are shown on Table 2 below.

**Table 2:** comparison of characteristics between the participants with and without circulatory failure, recruited from the Children Emergency Room of the University of Benin Teaching Hospital (Nigeria), from October 2018 to March 2019 (N=554)

Characteristics	Shock at presentation			
	No (%)	Yes (%)	c2	p-value
**Age (Years)**				
<1	184(84.0)	35(16.0)	2.641	0.267
1-5	166(85.6)	28(14.4)		
>5	119(90.2)	13(9.8)		
Median age(IQR)	60 (24-120)	66(24-132)	0.222†	0.824
**Source of patient referral**				
Private	32(69.6)	14(30.4)	8.967	0.011*
Public	36(80.0)	9(20.0)		
Home	313(86.2)	50(13.8)		
**Duration of illness (Days)**a				
<3	139(87.4)	20(12.6)	0.663	0.415
>=3	310(84.7)	56(15.3)		
Median (IQR)	4(2-7)	4(2-7)	0.594†	0.552
**Maternal educational level**				
None	51(75.0)	17(25.0)	14.031	0.001*
Primary	111(82.8)	23(17.2)		
Secondary	215(91.5)	20(8.5)		
**Pre-hospital care**				
None	279(86.1)	45(13.9)	19.945	<0.001*
ORSb	26(63.4)	15(36.6)		
Herbal concussion	10(83.3)	2(16.7)		
Others	160 (90.4)	17(9.6)		
**Gender**				
Male	246(84.8)	44(15.2)	0.148	0.701
Female	215(86.0)	35(14.0)		
**Socio-economic class**				
Upper	111(98.2)	2(1.8)	26.034	<0.001*
Middle	148(87.6)	21(12.4)		
Lower	160(77.7)	46(22.3)		

*significant at P < 0.05; Mann whitney u test; aDuration before admission; bORS = oral rehydration salts

**Multivariate logistic regression analysis for circulatory failure by selected clinical-demographic characteristics:** Table 3 shows multivariate logistic regression for cardiovascular shock by selected variables; children referred from private clinics were at least two times more likely to arrive CHER in shock compared to those coming directly from home (OR = 2.67, 95% CI: 1.07-6.69; p = 0.036). Children that received ORS as pre-hospital care were significantly more likely to present in shock in CHER (OR = 6.63, 95% CI: 2.15-20.46; p = 0.001). Children from lower socio-economic class families presented more frequently with shock than those from higher class (OR = 14.39, 95% CI: 2.61-79.44; p = 0.002). Maternal level of education and child´s duration of illness were not independent risk factors of cardiovascular shock in the participants, p > 0.05.

**Table 3:** multivariate logistic regression analysis for circulatory failure by selected clinical-demographic characteristics of participants, recruited from the Children Emergency Room of the University of Benin Teaching Hospital (Nigeria), from October 2018 to March 2019 (N=554)

Characteristics	Frequency (%)	aO.R	95% C.I.	p-value
**Source of patient**				
Private clinics	14(30.4)	2.67	1.07-6.69	0.036*
Public hospitals	9(20.0)	1.88	0.69-5.10	0.216
Home	50(13.8)	1.00		
**Mother level of education**				
None	17(25.0)	1.05	0.36-3.04	0.935
Primary	23(17.2)	0.83	0.33-2.06	0.689
Secondary	20(8.5)	1.00		
**Pre-hospital care**				
None	45(13.9)	1.38	0.62-3.04	0.428
ORS	15(36.6)	6.63	2.15-20.46	0.001*
Herbal concoction	2(16.7)	1.46	0.12-17.41	0.764
Others	17(9.6)	1.00		
**Socio-economic class**				
Upper	2(1.8)	1.00		
Middle	21(12.4)	8.26	1.68-40.56	0.009*
Lower	46(22.3)	14.39	2.61-79.44	0.002*

* Significant p-value; aOR = Adjusted odd ratio; CI = confidence interval; ORS = oral rehydration salts

## Discussion

This study shows some baseline characteristics of children presenting with circulatory failure in the emergency room; they had lower maternal levels of education than others without circulatory failure, consistent with prior reports of delayed treatment of ill children among less educated parents [13,14]. Maternal education apparently aids earlier recognition of worsening illnesses in children which can enhance prompt presentation to health facilities. Also, education can improve maternal decision-making and finance ensuring prompt access to paediatric emergency services in fee-paying health facilities [21,22]. Nevertheless, Abdulkadir *et al*. [23] in north-central Nigeria found that mothers´ secondary level of education was associated with late presentation of children to their emergency unit, perhaps related to their use of other community-based treatment options in the setting. In our study, children from high socioeconomic class (SEC) were less likely to present in circulatory failure in the emergency room, apparently due to their improved finance and health knowledge, consistent with earlier reports of good health-seeking behavior among families upper wealth quartiles [22,23]. However, there was no definite association between the age of the participants and the presence of shock, neonates being excluded from our study; neonatal infants are particularly vulnerable to shock because of their relatively high body surface area, insensible water loss and immature kidneys [11,21]. In contrast, Vekaria-Hirani *et al*. [10] in Kenya found that septic shock was commoner in the younger age groups with infants having the highest case fatality rates, apparently due to a majority of their participants being infants, including neonates. Moreover, the duration of the illnesses before admission in CHER as well as gender was not significantly associated with shock in this study; this may be due to the overall shortness of disease duration among our participants. Nonetheless, Oluwafemi *et al*. [12] and Isezuo *et al*. [24] in southern and northern Nigeria respectively found that late presentation to emergency units was associated with complications and poor outcomes among acutely-ill children.

The quality of care and referral threshold of referring hospitals as well as the expertise of transport team can influence the severity of illness at presentation in a tertiary centre [10,25]. In this index study, the source of referral was significantly associated with the presence of shock at presentation; children arriving from private clinic were twice more likely to present with circulatory failure in CHER. This could be a reflection of the severity of their illnesses before presenting at the referring clinic. Also, undue delays in transferring to a higher level of care may be present, especially when there are financial constraints, necessitated by out-of-pocket payment for healthcare. This is similar to findings by Royal *et al*. [26] in South Africa that critically-ill children transferred to tertiary care were unstable at arrival, possibly resulting from sub-optimal in-transit care, transport delays or illness progression. This highlights the need for efficient transport and retrieval services in our setting, comparable to those obtainable in developed countries, ensuring optimal stabilization of patients during transfer [25,27].

Furthermore, quality of prehospital care can affect the severity and outcome of childhood illnesses. Although some alternative medicines can have significant adverse effects on children, use of herbal therapies is common in African countries [15,16]. Nearly one third of our participants received pre-hospital care, and 2.2% of them took herbal concoctions before presentation in CHER. Likewise, in North-central Nigeria, Adeboye *et al*. [28] reported the use of several forms of traditional applications in 11.4% of paediatric emergencies and a majority of the caregivers believed that such applications cannot be used with conventional medicine, promoting late presentation to health facilities. Surprisingly, our participants who received oral rehydration salts (ORS) as prehospital care were more likely to present with shock in CHER, perhaps related to other levels of delays in accessing healthcare as well as presence of shock before the commencement of ORS. Also, wrongly prepared ORS can predispose to hyponatriemic or hypernatriemic dehydration and shock [29]. Hence, this finding should not be interpreted to undermine the usefulness of ORS in preventing dehydration in children. There is a need for regular education of caregivers on correct ORS preparation and adequate rehydration of children. The strengths of this study include its large sample size ensuring adequate power and the inclusion of all eligible children during the study period as well as the similar median ages and durations of illness (between children with and without shock) enabling subgroup comparison. Nonetheless, we acknowledge the limitation of cross-sectional study design adopted in this study, being unable to establish causal relationship. A cohort study design would have been able to compute the relative risk of circulatory failure associated with various pre-hospital therapies. Also, caregivers may not accurately recall all treatment history before presentation in the emergency setting, allowing unidentified confounders to limit the generaliseability of our findings.

## Conclusion

Potentially modifiable factors including quality of prehospital care, maternal educational level and parental socio-economic class influence the presence of shock in children seen in emergency departments. There is a need to develop appropriate pre-hospital care services and strengthen our limited retrieval/ emergency medical services. Also, expanding the national health insurance scheme coverage to include all households in low wealth quintiles is desirable, minimizing finance-related delays in accessing healthcare.

### What is known about this topic

- Several disease-related factors predispose children to circulatory failure;
- Low parental educational level leads to poor health-seeking behavior in many sub-Saharan African countries.

### What this study adds

- Lower parental socio-economic class is associated with circulatory failure in children seen at CHER in the setting;
- Prior treatment in a private clinic is associated with the presence of circulatory failure on admission at the tertiary centre;
- Pre-hospital care with oral rehydration salts is not protective against circulatory failure in critically-ill children in the setting.
